# The Role of Epigenetic Changes in the Progression of Alcoholic Steatohepatitis

**DOI:** 10.3389/fphys.2021.691738

**Published:** 2021-07-16

**Authors:** Hyeong Geug Kim, Jung-hyo Cho, Jeongkyu Kim, Seung-Jin Kim

**Affiliations:** ^1^Department of Biochemistry and Molecular Biology, Indiana University School of Medicine, Indianapolis, IN, United States; ^2^Department of East & West Cancer Center, Daejeon Korean Medicine Hospital of Daejeon University, Daejeon, South Korea; ^3^Department of Life Science, Chung-Ang University, Seoul, South Korea; ^4^Kangwon Institute of Inclusive Technology, Kangwon National University, Chuncheon, South Korea

**Keywords:** alcoholic steatohepatitis, epigenetics, sirtuins, DNA methylation, miRNAs

## Abstract

Alcoholic steatohepatitis (ASH) is a progression hepatitis with severe fatty liver and its mortality rate for 30-days in patients are over 30%. Additionally, ASH is well known for one-fifth all alcoholic related liver diseases in the world. Excessive chronic alcohol consumption is one of the most common causes of the progression of ASH and is associated with poor prognosis and liver failure. Alcohol abuse dysregulates the lipid homeostasis and causes oxidative stress and inflammation in the liver. Consequently, metabolic pathways stimulating hepatic accumulation of excessive lipid droplets are induced. Recently, many studies have indicated a link between ASH and epigenetic changes, showing differential expression of alcohol-induced epigenetic genes in the liver. However, the specific mechanisms underlying the pathogenesis of ASH remain elusive. Thus, we here summarize the current knowledge about the roles of epigenetics in lipogenesis, inflammation, and apoptosis in the context of ASH pathophysiology. Especially, we highlight the latest findings on the roles of Sirtuins, a conserved family of class-III histone deacetylases, in ASH. Additionally, we discuss the involvement of DNA methylation, histone modifications, and miRNAs in ASH as well as the ongoing efforts for the clinical translation of the findings in ASH-related epigenetic changes.

## Introduction

Alcohol consumption is the leading cause of alcoholic liver diseases (ALDs) worldwide, and it is generally accepted that obesity exacerbate its progression (Parker et al., 2018, 2019; Hwang et al., 2020). ALDs cover a wide range of diseases with various pathological spectrums including simple steatosis, alcoholic steatohepatitis (ASH), progressive liver fibrosis, alcoholic cirrhosis, and hepatocellular carcinoma (HCC) (European Association for the Study of Liver, 2012; Abenavoli et al., 2016). ASH corresponds to the intermediate stage between simple steatosis and progressive liver fibrosis, and its pathophysiological features are mainly characterized by inflammation along with lipid accumulation in the liver (Parker et al., 2018, 2019; Teschke, 2018). In humans, genome-wide association studies conducted in multiple groups have shown that some genetic modifiers, such as PNPLA3, TM6SF2, and MBOAT7 appear to play a pivotal role in ALD progression, however, there are limited studies and data supporting this association in ASH patient samples (Boccuto and Abenavoli, 2017; Meroni et al., 2018). To comprehend the relationship between alcohol consumption and obesity *in vivo*, Dr. Bin Gao’s group in NIAAA developed a rodent model of ASH though the high-fat diet (HFD)-plus-binge ethanol challenge, which mimics the concurrent chronic over-nutrition and excessive alcohol consumption in humans. Consequently, they found that over-nutrition and alcohol-drinking synergistically upregulate hepatic and serum Chemokine (*C-X-C* motif) ligand 1 (CXCL1) levels (Chang et al., 2015; Hwang et al., 2020). Interestingly, an earlier study had reported that chronic upregulation of chemokines such as CXCL2 is mediated through epigenetic mechanisms, consisting of DNA methylation and histone modification (Kiguchi et al., 2014). Collectively, these observations indicate that over-nutrition and alcohol-drinking synergistically promote ASH through induction of epigenetic genes. Accordingly, in this review we address the roles of epigenetic changes in ASH, whereby the mechanisms underlying ASH progression may be better appreciated to identify a therapeutic target.

## Alcoholic Steatohepatitis (ASH)

In general, ALD is a progressive pathological status generally starting with alcoholic fatty liver disease and progressing to ASH, which is accompanied with inflammation and liver injury. Chronic ASH can lead to liver fibrosis and cirrhosis, and finally to HCC (Seitz et al., 2018; Gao et al., 2019). Among the heavy alcoholic populations, approximately 20% of them are undergoing ASH condition by evidence of liver biopsy (Stickel and Seitz, 2010); however, no therapeutic currently exists for the treatment of ASH, and liver transplantation is the only treatment strategy in the clinic (Teschke, 2018). Although the exact definition of heavy alcohol users according to each institute, NIAAA normally defines it gender specific for men, more than four drink daily or 14 drinks weekly and three drinks daily or seven drinks weekly for women. On the other hand, SAMHSA mentions people who drinks binge on 5 or more days during last a month^1^. Generally, the etiology of ASH is thought to involve various causes, including genetic, epigenetic, and non-(epi)genetic factors, which considerably result in the inter-individual variation in ALD phenotype (Anstee et al., 2016). The efforts to elucidate the pathological mechanisms of ASH during the last decades (Ishak et al., 1991; Jmelnitzky, 1995; Stickel and Seitz, 2010; Rehm et al., 2013; Osna et al., 2017; Teschke, 2018) have shown that the associated ALD is mainly featured by hepatic steatosis, oxidative stress, toxicity due to the production of acetaldehyde, upregulation of inflammatory cytokines, and activation of the chemokine-induced inflammation deriving from the innate immune response (Ishak et al., 1991; Gao and Bataller, 2011; Dunn and Shah, 2016; Osna et al., 2017). Linking these findings may help elucidate the entire pathological steps of ASH, whereby relevant therapeutic approaches can be identified. Thus, we here aim to identify the etiological factors of ASH, especially focusing on epigenetic regulators, which can modulate pathophysiological pathways.

### Sirtuins

Sirtuins (Sirts) are a family of seven silent information regulator 2 proteins (Sirt 1–7), which are a group of Class III histone or protein deacetylases via regulation of nicotinamide adenine dinucleotide (NAD^+^)-dependent mode. Sirts mainly regulate cellular protein functions via various posttranslational modifications in differently located subcellular levels. Studies have reported that each type of sirtuin has a different subcellular localization pattern, thus sirtuin family members are critical epigenetic regulators to control energy metabolism, glucose transport and metabolism, and lipid regulations in the liver tissue under the NASH and ASH conditions (Dong et al., 2021). Sirt1 is localized in the nucleus or cytoplasm, being associated with metabolic disorders and inflammation relying on deacetylase activities (Yeung et al., 2004; Lawrence, 2009; Franceschi and Campisi, 2014). The NAD^+^-dependent deacetylase, Sirt2 is a cytoplasmic enzyme and mainly takes part in the cell cycle and tumorigenesis (Dryden et al., 2003; Vaquero et al., 2006; North and Verdin, 2007; Wang et al., 2020). Sirt3 is a soluble protein with NAD^+^-dependent deacetylase activity and exists in the mitochondria. It can regulate metabolic processes, especially the redox homeostasis (Zhang J. et al., 2020). Along with Sirt3, Sirt4 is also observed in the mitochondria and functions as a mitochondrial ADP-ribosyltransferase. Sirt4 suppresses insulin secretion by inhibiting mitochondrial glutamate dehydrogenase (GDH)-1 (Min et al., 2018; Liu et al., 2019). Sirt5 is also located in the mitochondria and shows deacetylase, desuccinylase, and demalonylase activities, which remove acetyl, succinyl, and malonyl groups from the lysine residues of proteins, respectively. Therefore, Sirt5 may regulate the urea cycle, which is involved in the deacetylation of cytochrome c, and energy metabolism (Polletta et al., 2015; Sun et al., 2016; Zhang Y. et al., 2017; Chiba et al., 2019). Sirt6 located in the nucleus is a chromatin-associated protein. It is implicated in the modulation of the metabolism, DNA repair, and inflammation (Geng et al., 2020; Klein et al., 2020; Onn et al., 2020). The last member of sirtuins, Sirt7, is localized in the nucleus and its mono-ADP-ribosyltransferase activity regulates the functions of intracellular regulatory proteins. This sirtuin can be required for ribosomal-DNA transcription (Tsai et al., 2012; Mohrin et al., 2015). Contrary to the actively researches of NASH in liver specific roles, ASH studies are rarely reported till recently. This may be attributed by the similarities of phenotypes between NASH and ASH, and most of patients did not mention about ALD; however, the specific roles of sirtuins on NASH and ASH are relatively discerned according to the specific molecular targets.

### Sirtuins and ASH

Although an exact role of sirtuins in ALD remains unclearly, previous studies suggested their roles on the inflammation, oxidative stress, and lipid metabolism, respectively. Hepatocyte specific abrogation of Sirt1 aggravates hepatic inflammation and steatosis during ALD especially owing to lipid metabolism dysregulation, modification of lipin-1 function (Yin et al., 2014). Sirt1 also leads to alter SREBP-1c by decrease of hyperacetylation levels (You et al., 2008). Besides, it exerts to reduce H3K9Ac by targeting to the lysine residues in nuclear factor-κB (NF-κB) (Purushotham et al., 2009; Shimazu et al., 2010). Additionally, Sirt1 hepatic deletion mice showed severe inflammatory reactions by Kupffer activation through Sirt1 failed to repress NFATc4 activities (Yin et al., 2014). In NASH, Sirt1 not only inactivates KCs cell inactivation, but also leads to lipolysis by increase of β-oxidation (Vilà et al., 2014). It also inactivates SREBP-1, SCD1 levels, and LKB1, but activates AMPK levels (Cheng et al., 2017).

Contrary to the beneficial effects of Sirt1, hepatic Sirt3 is known to aggravate ALD status during chronic ethanol fed mice model by up-regulation of Sirt3 expression levels. Especially, liver specific Sirt3 knocking-down mice led to ameliorations of ASH condition by improvement of autophagy, especially increases of LC3B II in hepatic protein levels. Accordance with the above outcomes, Sirt3 mediated autophagy induction condition of ethanol treated hepatocyte cells, AML 12 cells, showed cytoprotective effects on ethanol treated condition by inhibition of pro-apoptotic proteins Bax, but activation of Bcl2, respectively (Ma et al., 2019). During NASH by MCD diet fed mice model, hepatic Sirt3 is involved lipotoxicity mediated apoptosis by acetylation and mitochondrial respiratory complexes III and IV inactivation and regulates SOD2 activities (Kendrick et al., 2011; He et al., 2013). The roles of Sirt5 in ALD, previous study reported that hepatic protein levels of Sirt5 were depleted by 4 weeks of alcohol diet of wild type SD rat model. This condition allowed to communication between mitochondria and nucleus levels by evidences of hypophosphorylation of FoxO1, hyperacetylation of p53, and mitochondrial PGC-1a, respectively (Ma et al., 2019).

**FIGURE 1:**
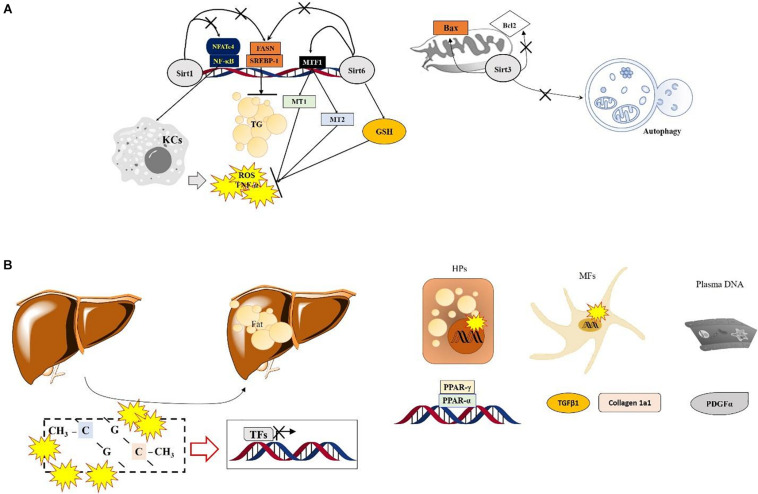
Specific roles of Sirtuins and DNA methylation in ASH. **(A)** Numerous SIRT-modulated mechanisms mainly regulate hepatic inflammation via inactivation of Kupffer cells and reduce lipogenesis during ASH. Sirt6 shows protective effects by normalizing the ethanol metabolism and increasing antioxidant components via enhancement of MTF1 acylation in the nucleus. Sirt3 enhances the mitochondrial apoptotic signals and prevents autophagy. **(B)** The role of DNA methylation in ASH. Lipid-droplet accumulation in the liver during ASH is suppressed by regulating factors associated with the lipid homeostasis or extracellular matrix via the Ufmylation pathways in hepatocytes and myofibroblasts. In plasma DNA, DNA methylation also regulates PDGFα expression during ASH.

Hepatic tissue specific deletion of Sirt6 in mice which was applied *Mx1-Cre* (for all liver cell types KO conditions) causes severe ALD by increasing hepatic oxidation and inflammation, which are regulated by hepatic metallothionein 1 and 2 (Mt1 and Mt2). Conversely, over expression of Sirt6 in the liver prevents the above-mentioned pathological alterations by enhancing cellular antioxidant activities and anti-inflammatory reactions, accompanied by upregulation of MT1/2. Sirt6 not only synergistically increases both Mt1 and Mt2 levels during the physiological status but also modulates hepatic MTs through controlling MTF1-induced acetylation (Kim et al., 2019; Figure 1A). On the other hand, liver tissue specific KO condition of Sirt6 activates deacetylation of Smad3 at lysine residues K333 and K378 in HSCs, during liver fibrosis development (Zhong et al., 2020).

## DNA Methylation

DNA methylation is well documented in the progression of ASH due to its specific role of epigenetic regulator to modulate transcription factors (TFs) as well as chromatin accessibility. DNA methylation, such as 5-Methylcytosine (5mC) and 5-hydroxymethylcytosine (5hmC), which are located near promoters with no or low transcriptional activity (Bird, 2002). DNA methylation has been uncovered to serve as the molecular basis of ALD (Zeybel et al., 2015).

Upon the function of the DNA methylation during ALD, it causes an alteration in the methionine metabolism via changes in the expression of various genes; however, the role of epigenetic machinery still remains unclearly. As major epigenetic target molecules of DNA modification both 5mC and 5hmC lead to modification of gene expression by regulating of TFs, mainly suppress certain genes transcription levels (Gopalakrishnan et al., 2008; Argentieri et al., 2017).

DNA methylation-related epigenetic regulation in the biopsy samples from ASH patients were severely modified by DNA methyltransferase 1 (DNMT1) and DNMT3B through increasing genomewide 5mC methylation level (Liu et al., 2014). One of considerable mechanism in linking DNA methylation and ASH is UFMylation, a biological process that attaches ubiquitously expressed small ubiquitin fold modifier 1 (UFM1), the most recently identified ubiquitin-like molecule, to target proteins (Wei and Xu, 2016). Although its particular role in ALD is not clearly revealed, the Ufmylation levels were lowered in ASH and this modification was led by increases in DNA methylation levels on promoter CpG of Ufm1, Ufc1, and UfSP1, which are correlated with increased DNMT1 and DNMT3B mRNA levels in AH patients (Liu et al., 2014; Shen et al., 2015).

Plasma DNA methylation at the gene promoter of PPARγ was increased in ALD-provoked liver cirrhosis patients by hypermethylation manners and increased at the site of particular *loci* in PDGFα gene. Interestingly the above methylation levels of PPARγ were higher in hepatocyte than myofibroblast in hepatic fibrosis patients of ALD (Hardy et al., 2017). Regarding lipid homeostasis and fibrosis related genes including PPARα, PPARα, and TGFβ1, Collagen 1A1, PDGFα genes in ALD patients, contrary to NAFLD patients show different patterns especially increase of DNA methylation at the site of TGFβ1 exon was upregulated in CpG2, but reverse manner of Collagen 1A1 gene (Zeybel et al., 2015; Figure 1B).

## Histone Modifications

In addition to DNA methylation, histone modifications including histone methylation, acetylation, phosphorylation, and ubiquitination, etc., are also well known as a key epigenetic regulator. It is reported that several key metabolites are involved in each histone modification such as acetate, S-adenosylmethionine (SAM), NAD^+^, and zinc are relevant to alcohol metabolism, especially in ASH (Moghe et al., 2011). Herein, thus, we next focus on methylation, acetylation, phosphorylation, ubiquitination, and sumoylation of histone during ASH development via emphasis on their specific roles of epigenetic modulations, especially gene expression (Table 1).

**TABLE 1:** Summary of histone modifications and miRNAs involved in ASH.

**Type of epigenetic modification**	**Alteration upon exposure to alcohol**	**Targets altered by epigenetic changes**	**Effect upon exposure to alcohol**	**References**
**Histone methylation**	H3K4me↑	Adh↑, GST-yc2↑	Speed up ethanol metabolism; Progression of fatty liver, Inflammation and liver cirrhosis	Pal-Bhadra et al., 2007
	H3K9me↓	Lsdh↓, cytP4502c11↓	Progression of fatty liver, Inflammation and liver cirrhosis	Pal-Bhadra et al., 2007
	H3K9me↑	TNF-α↓	Exacerbation of the inflammatory response	Nagy, 2015
**Histone acetylation**	H3K9ac↑	TNF-α↑; PNPLA3↑	HAT activity and HDAC inhibition; exacerbation of the inflammatory response	Nagy, 2015; Restrepo et al., 2017
**Histone phosphorylation**	H3S10ph↑ H3S28ph↑	c-fos↑, c-jun↑; MAPK1↑	Transcriptional activation	Lee and Shukla, 2007; James et al., 2012
**miRNAs**	miR-155↑ miR-21↑	TNF-α↑, IL-1β↑, PPAR[mymaths]γ↓[mymathe]	Increase inflammation response and Steatosis	Hartmann and Tacke, 2016; Zhang T. et al., 2020
	miR-212↑	ZO-1↓	Increased gut and liver injury	Tang et al., 2015
	miR-122↑↓	HIF-1α↑; PGC1a↑; ApoE↑	Lipid synthesis, export and cholesterol homeostasis	Krützfeldt et al., 2005; Esau et al., 2006; Bala et al., 2012; McDaniel et al., 2014; Momen-Heravi et al., 2015

### Histone Methylation

Histone methylation is a dynamic process with important roles in development and differentiation (Kouzarides, 2007; Eissenberg and Shilatifard, 2010), which are being catalyzed by two groups of enzymes, histone methyltransferases (HMTs) and histone demethylases (HDMs), respectively. First, HMTs catalyze the transfer of methyl group(s) from the cofactor S-adenosyl-L-methionine (SAM), the primary methyl group donor, most heavily to lysine or arginine residue of histone H3 followed by that of histone H4, affecting in turn the recruitment and binding of several regulatory proteins to chromatin (Morera et al., 2016; Hyun et al., 2017; Kaniskan et al., 2018). According to the accumulated evidence revealed that ASH is deeply linked to decreased level of SAM in the liver tissues from rodents and non-human primates (Lieber, 2002; King et al., 2016), which suggest that the transfer of methyl group catalyzed by HMTs can be interrupted and ultimately affects global pattern of gene expression. Additionally, another study performed in rodents also reported that acute and chronic ethanol exposures altered the expression levels of several susceptible genes which are associated with site specific histone methylation at their regulatory regions by performance of both *in vitro* and *in vivo* experiments (Pal-Bhadra et al., 2007; Mandrekar, 2011). It should also be noted that ethanol consumption generally induced deleterious effect on the inflammatory response because it is well accepted that upon ethanol exposure one of a key pro-inflammatory cytokine, tumor necrosis factor-α (TNF-α) is silenced by H3K9 methylation (Nagy, 2015). Together, it is evident that mapping the pattern of histone methylation in response to alcohol exposure would help us better understanding the underlying mechanisms of ASH progression.

### Histone Acetylation

Global changes in histone modifications upon ethanol exposure suggest that they would be resulted in genome-wide alterations in gene expression; however, some cases for depending on types of alcohol exposures histone acetylation, is modulated by two types of specific enzymes such as histone acetyltransferases (HATs) and histone deacetylases (HDACs), can be limited to a type of subsets of genes. Indeed, an increased gene-selective levels of histone H3K9 acetylation in the liver tissue is observed by chronic ethanol feeding using rat model without global changes in histone acetylation at other lysine residues such as H3K14, H3K18, and H3K23, respectively (Park et al., 2003, 2012), pointing to an existence of disease relevant epigenetic signatures during the progression of ASH.

In addition, it is now generally accepted that in the liver, level of histone H3 acetylation and alcohol induced fatty liver depend on the balance between the activity of HATs and HDACs being affected by alcohol consumption in rat hepatocytes and patients with Rheumatoid arthritis and osteoarthritis (Ito and Adcock, 2002; Park et al., 2005; Huber et al., 2007). For example, ethanol-regulated HAT and GCN5 in human cell lines modulates the expression of PGC1b, a protein involving in fat metabolism (Kelly et al., 2009; Choudhury et al., 2011). Intriguingly, ASH condition of rat model leads to increases of another HAT called p300 levels in the cellular nuclei at a peak blood alcohol level, which is correlated with increased H3K9 acetylation activities (Bardag-Gorce et al., 2007). In the case of HDACs, aforementioned SIRT1 plays a pivotal role in the pathogenesis of ASH via impairments of SIRT1 and SIRT1-regulated genes encoding lipogenic or fatty acid oxidation enzymes (Yin et al., 2012). Accumulating evidences recommended that both HATs and HDACs are likely to play a role in ethanol-induced liver injury. This implies again that a certain type of features shown in histone acetylation can be used as biomarker of a certain disease such as ASH (Park et al., 2005; Shepard et al., 2008; Kirpich et al., 2012; Pochareddy and Edenberg, 2012).

### Histone Phosphorylation

Contrary to histone methylation and acetylation, histone phosphorylation is mediated by two types of enzymes including kinases and phosphates, having opposing modes of action, which works together with other histone modifications, thus generating the platform for mutual interactions between the modifications under the ASH condition. Under the acute ethanol exposure status, histone H3S10 and H3S28 phosphorylation which were depended on p38 mitogen-activated protein kinase (MAPK), are directly able to affect acetylation levels at two amino acid residues of the same histone H3K9 and H3K14 acetylation (Lo et al., 2000; Edmondson et al., 2002; Lee and Shukla, 2007; Aroor et al., 2010). Furthermore, H3S10 phosphorylation can induce transactivation by interaction with H4K16Ac, suggesting a synergistic relationship between phosphorylation and acetylation in transcription regulation on a subset of genes in response to alcohol exposure. For example, both histone H3S10 phosphorylation and the same histone H3K14 acetylation are known to be involved in cytokine-induced gene expression mediated by nuclear IKKα leading to NF-κB activation (Anest et al., 2003; Yamamoto et al., 2003). Intriguingly, retinoic acid receptor-β and c-jun gene regulation is not linked to histone H3 acetylation but histone H3 phosphorylation (Clayton et al., 2000; Thomson et al., 2001; Lefebvre et al., 2002) indicating that these two epigenetic changes can occur independently. Great effort is therefore needed to understand whether alcohol induces crosstalk between for instance histone phosphorylation and acetylation or as independent pathways to regulate target gene expression in ASH, because the various histone modifications observed in cultured hepatocytes in response to ethanol follow different time courses.

### Other Histone Modifications

Unlike other histone modifications as mentioned above, histone ubiquitination occurs in a variety of histones, including histone H1, H2A, H2B, and H3. Especially, mono-ubiquitination has a critical role in the translocation of proteins, DNA-damage signaling, and transcriptional regulation. H2A mono-ubiquitination is generally associated with gene silencing, whereas H2B mono-ubiquitination is correlated with transcriptional activation (Zhang, 2003). Similar to histone phosphorylation, histone mono-ubiquitination is known to induce acetylation of the same histone (Zhang X. et al., 2017). The effects of alcohol consumption on the histone ubiquitination in the liver have not been studied in detail. However, a previous study of transcriptional profiling of post-mortem brain samples from individuals with a history of alcohol abuse or dependence showed abnormalities in the ubiquitin signaling (Ishino et al., 2014). Accordingly, it could be legitimate to presume that alcohol exposure may exacerbate histone ubiquitination.

Histone sumoylation is another post-translational modification involved in diverse biological processes, including the stress response, protein stability, the cell cycle, apoptosis, nucleocytoplasmic transport, and transcriptional regulation. Histone sumoylation occurs on lysine residues and is known to be a transcriptionally repressive modification (Rubio et al., 2013). Small ubiquitin-like modifier (SUMO)-conjugated yeast histones appear to suppress gene expression by opposing active histone marks, such as acetylation or ubiquitylation (Nathan et al., 2006). Although only a few studies have explored how alcohol consumption affect histone sumoylation, a study has mentioned that the sumoylation in the livers of mice fed with ethanol is significantly reduced relative to the levels in the control mice (Nisticò et al., 2014). This finding implies alcohol-mediated changes in the level of histone sumoylation. Together, much more attention should be given to those modifications to better understand the complexity of epigenetic networks involved in the pathogenesis of ASH.

## Micrornas in ASH

In addition to histone modifications, miRNAs, regulator of post-transcriptional gene expression, are abundant in the liver and play key roles in diverse biological processes associated with liver injury, including hepatocyte regeneration, apoptosis, and inflammation (Schueller et al., 2018). It should be noted that the levels of a subset of miRNAs was found to be dysregulated upon alcohol consumption. Deregulation of the levels of some specific miRNAs has commonly been identified, suggesting the importance of these factors in alcoholic liver injury. Numerous reports on miRNA dysregulation in alcohol-induced liver diseases summarize the up- and down-regulated miRNAs in the liver in response to alcohol consumption (Table 1), and these differentially expressed miRNAs may act as causative factors of ASH. Although changes in miRNA levels can affect the expression of the enzymes involved in other epigenetic modifications, expression of miRNAs themselves can be subject to regulation via histone modifications that regulate miRNA expression. For example, ethanol upregulates miR-155 via the recruitment of a transcriptional activator called NF-κB to the miR-155 promoter (McDaniel et al., 2014), subsequently causing epigenetic changes associated with gene activation. MiR-155 is also known as a regulator of the hepatic secretion of inflammatory agents, such as lipopolysaccharide (LPS)-induced TNF-α, consistent with a pathogenic role in the liver in response to acute or chronic alcohol consumption (Bala et al., 2011). More recently, growing evidence suggests that circulating miRNAs can be used as stable biomarkers for alcohol-induced liver diseases because similar miRNA expression patterns are found both in the liver and blood of ALD patients. Moreover, miRNAs can also stay in the plasma in a stable form as well as in the blood of ALD patients, making these small RNAs feasible biomarkers for the detection of ALD and other liver disorders, suggesting that the effect of acute or chronic alcohol consumption on the liver may be defined by quantifying circulating miRNAs taken from serum or plasma. It has been therefore increasingly appreciated that understanding the role of miRNAs in ASH has many potential aspects that may be applied into novel therapeutic approaches in ASH. Although there is currently no miRNA-related treatment modality in ASH, miRNAs may be utilized as key therapeutic targets in ALD in the near future.

## Conclusion and Future Perspectives

**FIGURE 2:**
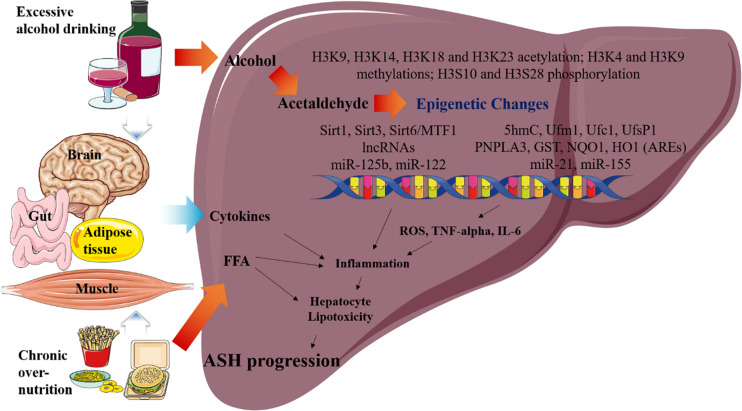
Overview of the epigenetic changes in ASH. Metabolic pathways regulated by acetaldehyde, cytokine, and FFA are induced by excessive alcohol drinking and chronic over- nutrition. Excessive alcohol drinking and chronic over-nutrition synergistically induces ASH progression via activating several different signaling pathways in the liver, including epigenetic changes. Additionally, hepatic cytokine and FFA levels of cytokines are induced as a result of the associated multiple organ damages, such as those in the adipose tissue, brain, intestines, and muscles. Eventually, this pathway increases hepatic inflammation and hepatocyte lipotoxicity, which result in the stimulation of ASH.

Over the past decade, an increased understanding of the relationship between epigenetic factors and ASH has been developed. Nevertheless, the specific epigenetic mechanisms underlying the pathophysiology of ASH still remain unclear. Thus, we addressed the important roles of the epigenetic regulators that can modulate the pathophysiological pathways in ASH. Particularly our study focuses on the following areas of current research on the epigenetic changes reported in ASH, mainly sirtuins, histone acetylation, methylation, and phosphorylation. These epigenetic changes increase cytokine activation, inflammation, and lipogenesis in the liver, eventually leading to ASH progression (Figure 2). In the future, these epigenetic regulators as well as the ASH-related metabolic pathways may be targeted for the treatment of ASH. Moreover, further investigation of these epigenetic regulators may also lead to effective treatment modalities for metabolic diseases, including ALD.

## Author Contributions

HK designed and wrote the manuscript. J-HC critically revised the manuscript. S-JK and JK supervised the whole project and wrote the manuscript. All authors contributed to the article and approved the submitted version.

## Conflict of Interest

The authors declare that the research was conducted in the absence of any commercial or financial relationships that could be construed as a potential conflict of interest.

## Footnotes

1 https://www.niaaa.nih.gov/alcohol-health/overview-alcohol-consumption/moderate-binge-drinking

## Abbreviations

ALD: alcoholic liver disease
ASH: alcoholic steatohepatitis
CXCL1: chemokine (C-X-C motif) ligand 1
DNMT1: DNA methyltransferase 1
GDH: glutamate dehydrogenase
HAT: histone acetyltransferases
HCC: hepatocellular carcinoma
HDACs: histone deacetylases
HDMs: histone demethylases
HFD: high-fat diet
HMTs: histone methyltransferases
LPS: lipopolysaccharide
MAPK: mitogen-activated protein kinase
miRNA: microRNA
MT: metallothionein
NASH: non-alcoholic steatohepatitis
ncRNAs: non-coding RNAs
NF-κB: nuclear factor κB
PPAR γ: peroxisome proliferator-activated receptor gamma
SAM: S-adenosyl-L-methionine
SIRT: sirtuin
SUMO: small ubiquitin-like modifier
TFs: transcription factors
TNF-α: tumor necrosis factor-α
5hmC: 5-hydroxymethylcytosine
5mC: 5-Methylcytosine.
